# Primed Immune Responses Triggered by Ingested Bacteria Lead to Systemic Infection Tolerance in Silkworms

**DOI:** 10.1371/journal.pone.0130486

**Published:** 2015-06-24

**Authors:** Atsushi Miyashita, Shinji Takahashi, Kenichi Ishii, Kazuhisa Sekimizu, Chikara Kaito

**Affiliations:** Laboratory of Microbiology, Graduate School of Pharmaceutical Sciences, The University of Tokyo, 3–1, 7-chome, Hongo, Bunkyo-ku, Tokyo, 113–0033, Japan; University of Tours, FRANCE

## Abstract

In the present study, we examined whether microorganisms collaterally ingested by insects with their food activate the innate immune system to confer systemic resistance against subsequent bacterial invasion. Silkworms orally administered heat-killed *Pseudomonas aeruginosa* cells showed resistance against intra-hemolymph infection by *P*. *aeruginosa*. Oral administration of peptidoglycans, cell wall components of *P*. *aeruginosa*, conferred protective effects against *P*. *aeruginosa* infection, whereas oral administration of lipopolysaccharides, bacterial surface components, did not. In silkworms orally administered heat-killed *P*. *aeruginosa* cells, *P*. *aeruginosa* growth was inhibited in the hemolymph, and mRNA amounts of the antimicrobial peptides cecropin A and moricin were increased in the hemocytes and fat body. Furthermore, the amount of paralytic peptide, an insect cytokine that activates innate immune reactions, was increased in the hemolymph of silkworms orally administered heat-killed *P*. *aeruginosa* cells. These findings suggest that insects sense bacteria present in their food by peptidoglycan recognition, which activates systemic immune reactions to defend the insects against a second round of infection.

## Introduction

Insects have hard exoskeletons that protect them against microbial invasion. The insect digestive tract, however, continuously comes into contact with various microorganisms present in the foods they ingest [1–4]. The intestinal epithelia recognize pathogenic microorganisms and activate an innate immune system in which the Imd and Toll pathways play central roles [5–9]. For example, in the fruit fly *Drosophila melanogaster*, peptidoglycan recognition protein-LC (PGRP-LC) in the intestinal epithelial cells recognizes Gram-negative peptidoglycans and activates the Imd pathway to produce antimicrobial peptides in the gut [10–12]. The production of antimicrobial peptides and reactive oxygen species is important for the fly’s resistance against oral bacterial infection [13, 14]. In *Bombyx mori* silkworm larvae, oral administration of *Escherichia coli* induces the production of antimicrobial peptides in the gut [15]. These findings indicate that activation of immune reactions in the gut functions to defend insects against oral infection by ingested pathogenic bacteria. Oral infection also induces the production of antimicrobial peptides in tissues other than the gut in *D*. *melanogaster* [16–18]. The physiological significance of the ectopic production of antimicrobial peptides outside the gut is unclear.

Insect foods, such as plants and small-sized animals, often contain many bacteria. Insects with open wounds in such environments are thought to be at high risk for invasion by bacteria into the hemolymph. Therefore, the ability to protect themselves against the invasion of pathogenic bacteria from the food supply into the hemolymph is advantageous. In addition, insects have primed immune responses, in which a sublethal infection induces infection tolerance against a second round of infection [19–22]. We recently revealed that silkworms have a primed immune system that recognizes bacterial peptidoglycans and confers persistent infection resistance by increasing the production of antimicrobial peptides [23]. Based on these findings, we hypothesized that systemic activation of immune reactions induced by microorganisms in the silkworm food confers resistance against a second round of bacterial invasion in the hemolymph.

Silkworms eat a constant daily amount of an artificial diet, allowing for quantitative peroral administration of samples [24, 25]. The body size of the silkworms is sufficient to inject samples quantitatively and thus silkworms are suitable for evaluating infection resistance by quantitative parameters [26]. Furthermore, individual tissues, such as the gut, fat body, and hemocytes, can be isolated to measure gene expression [27]. Here, we examined whether oral administration of heat-killed bacteria confers resistance to subsequent infection in the silkworm hemolymph.

## Results

### Oral administration of heat-killed *P*. *aeruginosa* induced infection resistance of silkworms against *P*. *aeruginosa*

**Fig 1:**
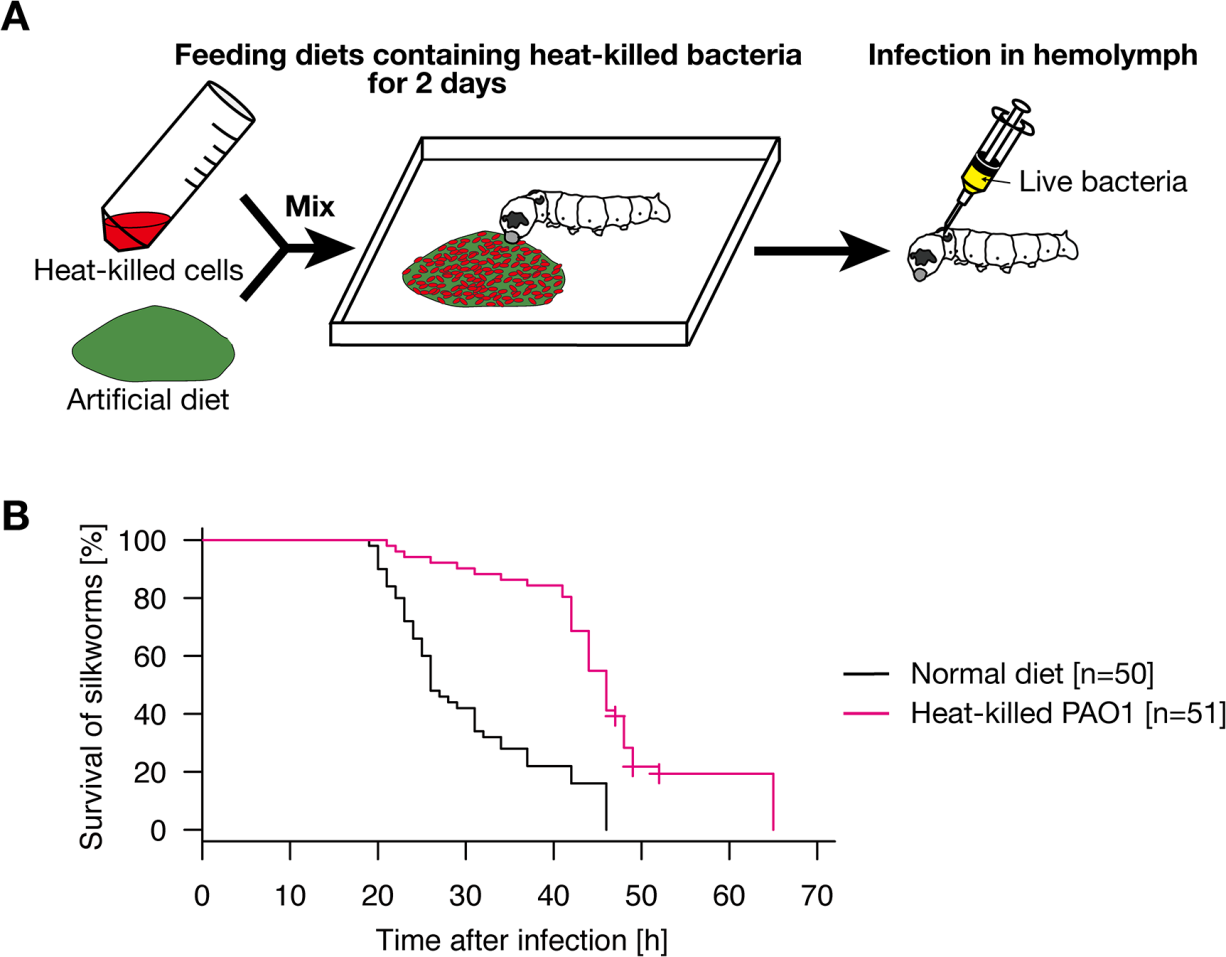
Resistance of silkworms orally administered heat-killed *P*. *aeruginosa* cells against *P*. *aeruginosa* infection. A. Experimental scheme of the study. Heat-killed bacterial cells were mixed with artificial diet and administered to silkworms for 2 d. Live bacteria were then injected into the silkworm hemolymph. B. Silkworms were fed a diet containing heat-killed *P*. *aeruginosa* cells (heat-killed PAO1 diet, n = 51) or a normal diet (n = 50) for 2 d and then injected with *P*. *aeruginosa*. Results from five independent trials (S1 Table, exp.1-3 and 5–6) were combined into a single analysis. The combined survival curve is shown in the figure. Survival of silkworms fed the diet containing heat-killed *P*. *aeruginosa* was significantly higher than that of silkworms fed the normal diet (p = 4.59E-11). None of the mock-infected silkworms died (S1 Fig).

Silkworms were orally administered heat-killed *P*. *aeruginosa* for 2 d and then live *P*. *aeruginosa* were injected into the  hemolymph (Fig 1A). The results obtained from independent experimental replicates are presented in S1 Table and S1 Fig, and the combined survival data are presented in Fig 1B. Silkworms orally administered heat-killed *P*. *aeruginosa* survived longer than those not administered heat-killed *P*. *aeruginosa* (Fig 1B, p = 4.59E-11). All the saline-injected silkworms survived (S1 Fig), suggesting that the observed deaths were due to *P*. *aeruginosa* infection. These findings suggest that oral administration of heat-killed *P*. *aeruginosa* resulted in silkworm resistance against *P*. *aeruginosa* infection in the hemolymph.

**Fig 2:**
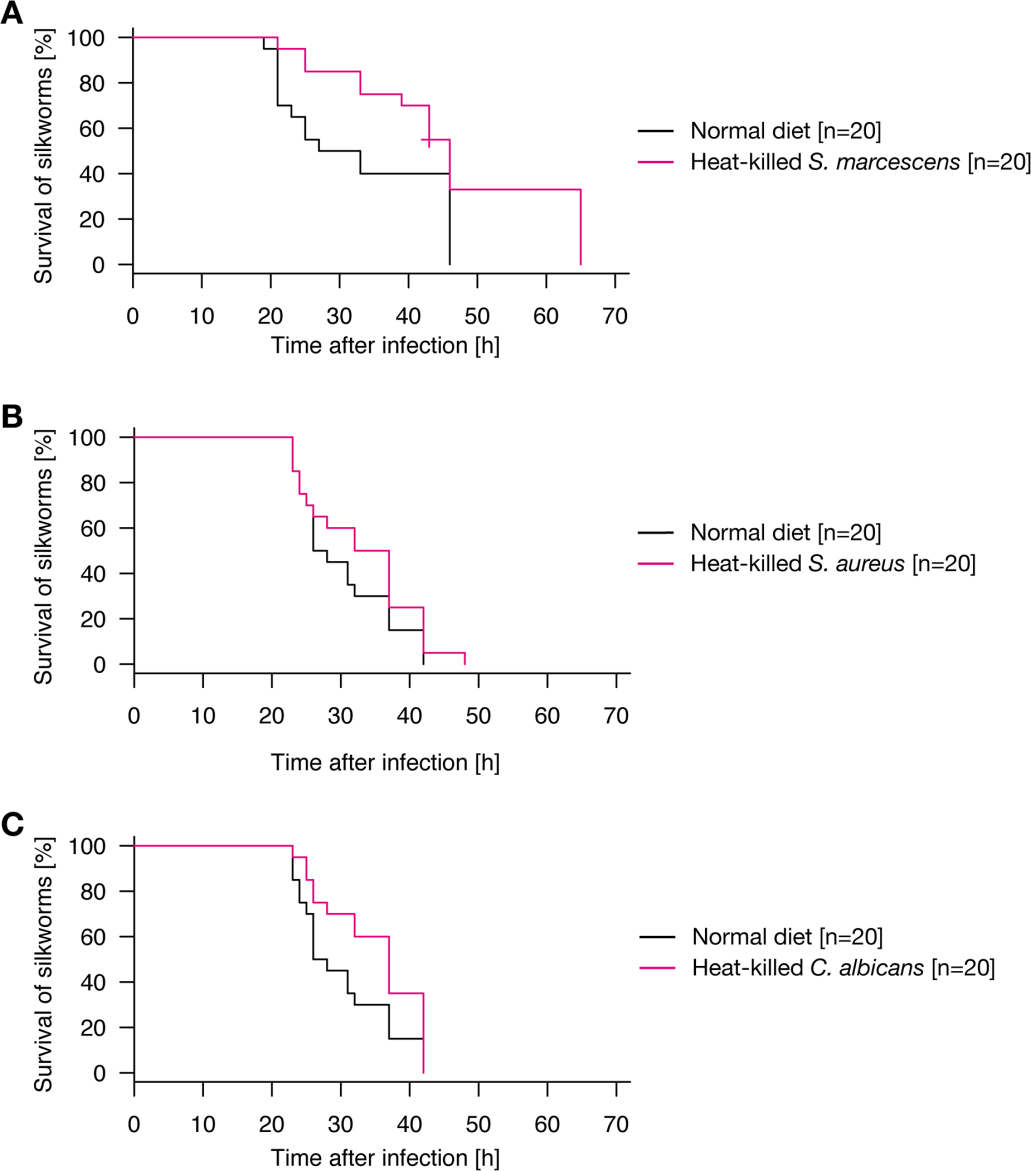
Resistance of silkworms orally administered with heat-killed cells of various microorganisms against *P*. *aeruginosa* infection. A. Silkworms were fed a normal diet (n = 20) or a diet containing heat-killed cells of *S*. *marcescen*s (n = 20) for 2 d, and then infected with *P*. *aeruginosa* in hemolymph. Results from two independent trials (S1 Table, exp. 3–4) were combined into a single analysis. The combined survival curve is shown in the figure. Survival of silkworms fed a diet containing heat-killed *S*. *marcescens* cells was significantly higher than that of silkworms fed a normal diet (p = 0.0219). None of the mock-infected silkworms died (S1 Fig). B. Silkworms were fed a normal diet (n = 20) or a diet containing heat-killed *S*. *aureus* cells (n = 20) for 2 d, and then injected with *P*. *aeruginosa*. Results from two independent trials (S1 Table, exp. 5–6) were combined into a single analysis. The combined survival curve is shown in the figure. Survival of silkworms fed a diet containing heat-killed *S*. *aureus* cells was not significantly different from that of silkworms fed a normal diet (p = 0.235). None of the mock-infected silkworms died (S1 Fig). C. Silkworms were fed a normal diet (n = 20) or a diet containing heat-killed *C*. *albicans* cells (n = 20) for 2 d, and then injected with *P*. *aeruginosa*. Results from two independent trials (S1 Table, exp. 5–6) were combined into a single analysis. The combined survival curve is shown in the figure. Survival of silkworms fed a diet containing heat-killed *C*. *albicans* cells was significantly higher than that of silkworms fed a normal diet (p = 0.0494). None of the mock-infected silkworms died (S1 Fig).

We then examined whether oral administration of heat-killed microorganisms other than *P*. *aeruginosa* confers infection resistance of silkworms against *P*. *aeruginosa* infection in the hemolymph. Silkworms that were first orally administered heat-killed *Serratia marcescens*, a Gram-negative pathogenic bacterium, survived longer than those not administered heat-killed *S*. *marcescens* when subsequently infected with *P*. *aeruginosa* (Fig 2A, p = 0.0219). In contrast, oral administration of heat-killed *Staphylococcus aureus*, a Gram-positive pathogenic bacterium, did not prolong the survival time of silkworms infected with *P*. *aeruginosa* (Fig 2B, p = 0.235). Oral administration of heat-killed *Candida albicans*, a pathogenic fungus, slightly prolonged the survival time of silkworms infected with *P*. *aeruginosa* (Fig 2C, p = 0.0494). These findings suggest that oral administration of heat-killed Gram-negative bacteria, as well as fungi, confers resistance against intra-hemolymph infection by *P*. *aeruginosa*.

**Fig 3:**
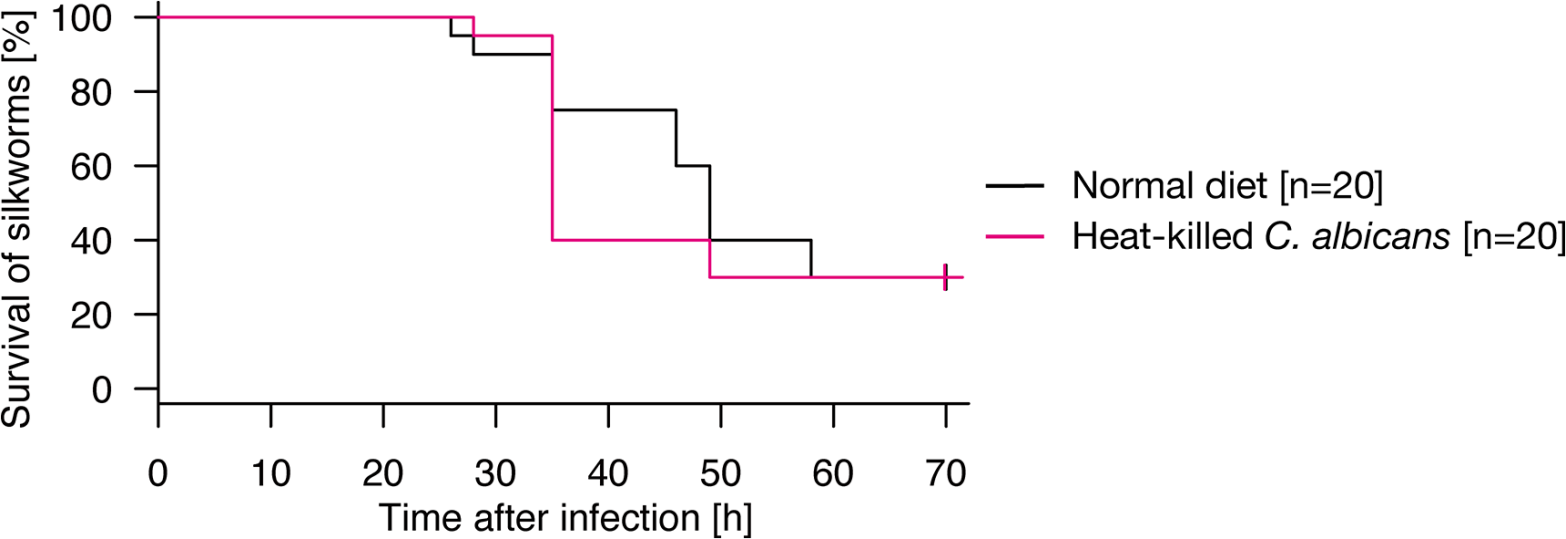
Resistance of silkworms orally administered heat-killed fungi against fungal infection. Silkworms were fed a normal diet (n = 20) or a diet containing heat-killed *C*. *albicans* cells (n = 20) for 2 d, and then injected with *C*. *albicans*. Results from two independent trials (S1 Table, exp. 7–8) were combined into a single analysis. The combined survival curve is shown in the figure. Survival of silkworms fed the diet containing heat-killed *C*. *albicans* cells was comparable to that of silkworms fed the normal diet (p = 0.995). None of the mock-infected silkworms died (S1 Fig).

We further examined whether the protective effect of heat-killed fungal cells are observed against fungal infection. Oral administration of heat-killed *C*. *albicans* did not prolong survival time when the hemolymph was infected with *C*. *albicans* (Fig 3, p = 0.995). This result suggests that oral administration of heat-killed fungi is not effective against subsequent intra-hemolymph fungal infection.

### Oral administration of *P*. *aeruginosa* peptidoglycans confers infection resistance against *P*. *aeruginosa*

**Fig 4:**
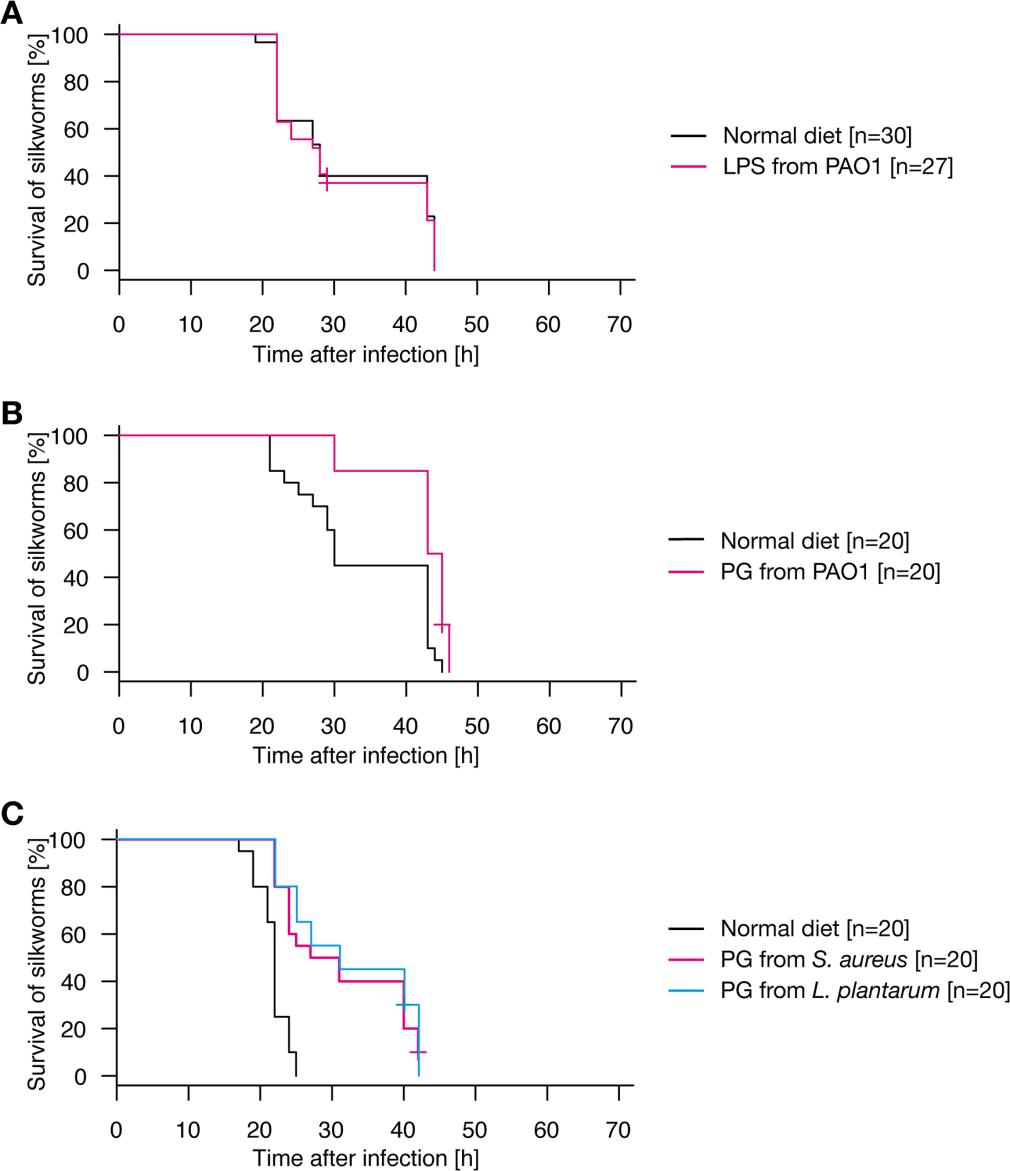
Oral administration of *P*. *aeruginosa* peptidoglycan leads to infection resistance of silkworms against *P*. *aeruginosa*. A. Silkworms were fed a normal diet (n = 30) or a diet containing *P*. *aeruginosa* lipopolysaccharides (n = 27) for 2 d and then injected with *P*. *aeruginosa*. Results from three independent trials (S2 Table, exp. 1–3) were combined into a single analysis. The combined survival curve is shown in the figure. Survival of silkworms fed the normal diet did not differ significantly from that of silkworms fed the diet containing lipopolysaccharides (p = 0.893). None of the mock-infected silkworms died (S2 Fig). B. Silkworms were fed a normal diet (n = 20) or a diet containing *P*. *aeruginosa* peptidoglycans (n = 20) for 2 d, and then injected with *P*. *aeruginosa*. Results from two independent trials (S2 Table, exp. 4–5) were combined into a single analysis. The combined survival curve is shown in the figure. The survival of silkworms fed the diet containing *P*. *aeruginosa* peptidoglycans was significantly longer than that of silkworms fed the normal diet (p = 1.76E-04). None of the mock-infected silkworms died (S2 Fig). C. Silkworms were administered a normal diet (n = 20), diet containing *S*. *aureus* peptidoglycans (n = 20), or diet containing *L*. *plantarum* peptidoglycans (n = 20) for 2 d, and then injected with *P*. *aeruginosa*. Results from two independent trials (S2 Table, exp. 6–7) were combined into a single analysis. The combined survival curve is shown in the figures. Survival of silkworms fed a diet containing *S*. *aureus* or *L*. *plantarum* peptidoglycans was significantly longer than that of silkworms fed a normal diet (p = 5.62E-06 or 3.37E-07). None of the mock-infected silkworms died (S2 Fig).

Lipopolysaccharides are abundantly present in Gram-negative cell walls, and peptidoglycans are present in both Gram-negative and Gram-positive bacterial surfaces. These molecules activate innate immune responses. Therefore, we examined whether oral administration of lipopolysaccharides or peptidoglycans confers resistance of silkworms against intra-hemolymph infection by *P*. *aeruginosa*. Silkworms orally administered *P*. *aeruginosa* lipopolysaccharides did not survive longer than silkworms not administered lipopolysaccharides when the hemolymph was infected with *P*. *aeruginosa* (Fig 4A, p = 0.893). In contrast, silkworms orally administered *P*. *aeruginosa* peptidoglycans survived significantly longer than silkworms not administered *P*. *aeruginosa* peptidoglycans when the hemolymph was infected with *P*. *aeruginosa* (Fig 4B, p = 1.76E-04). These findings suggest that peptidoglycans were responsible for the activity of heat-killed *P*. *aeruginosa* cells to confer resistance to the silkworms against *P*. *aeruginosa* intra-hemolymph infection.

Because oral administration of heat-killed *S*. *aureus* cells did not protect silkworms from *P*. *aeruginosa* infection, we examined whether the protecting effects are different between Gram-positive and Gram-negative peptidoglycans. We extracted peptidoglycans from *S*. *aureus* and *Lactobacillus plantarum*. *S*. *aureus* has a Gram-positive type peptidoglycan and *L*. *plantarum* has a DAP-type peptidoglycan containing diaminopimelic acid [28, 29]. Oral administration of both *S*. *aureus* peptidoglycan and *L*. *plantarum* peptidoglycan prolonged silkworm survival time when the hemolymph was infected with *P*. *aeruginosa* (Fig 4C, p = 5.62E-06 and 3.37E-07, respectively). The effects of *S*. *aureus* peptidoglycan and *L*. *plantarum* peptidoglycan were comparable (p = 0.944). These findings suggest that orally ingested peptidoglycans from *S*. *aureus* and *L*. *plantarum* confer infection resistance against *P*. *aeruginosa* infection in silkworm hemolymph.

### Induction of antimicrobial activity in the silkworm hemolymph by oral administration of heat-killed *P*. *aeruginosa* cells

**Fig 5:**
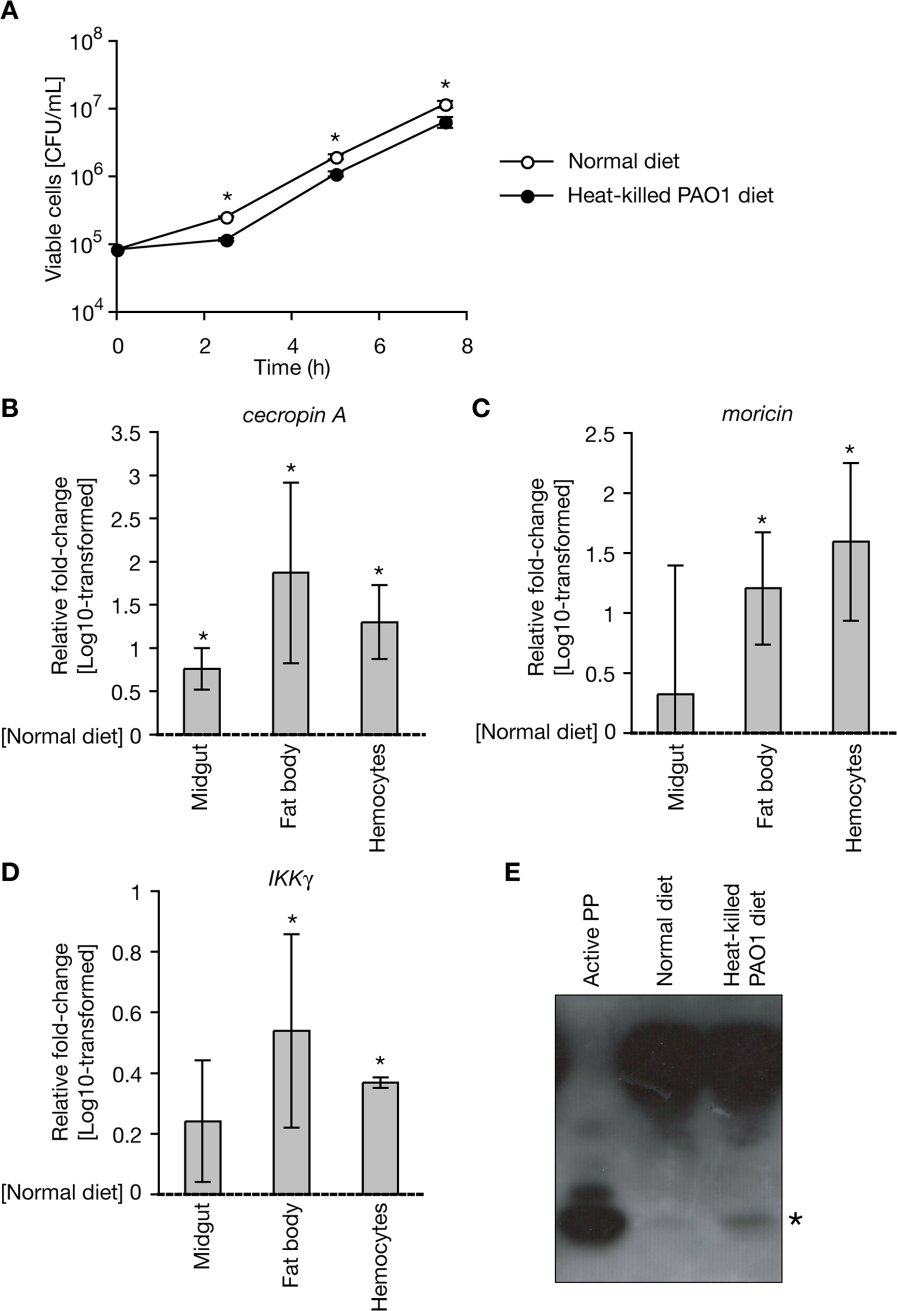
Activation of systemic immunity in silkworms by oral administration of heat-killed *P*. *aeruginosa*. A. Hemolymph samples were collected from fifth instar silkworms (n = 3) fed a diet containing heat-killed *P*. *aeruginosa* cells for 23 h, and living *P*. *aeruginosa* was inoculated into the hemolymph and incubated at 37°C. After incubation, samples were diluted and spread on agar plates for measurement of viable cell numbers. Data are shown as mean ± SD. Asterisk indicates p < 0.05 (Student’s t-test). A representative result from two independent experiments is shown. B, C, D. Total RNAs were extracted from the midgut, fat body, or hemocytes of fifth instar silkworms fed diet containing heat-killed *P*. *aeruginosa* cells for 2 d. The amounts of cecropin A, moricin, and IKKγ mRNA in the extracted RNA fractions were then measured. Each RNA amount was normalized by the mRNA amount of elongation factor-2. Values in the silkworms fed with heat-killed *P*. *aeruginosa* cells relative to that in the silkworms fed with normal diet were log-transformed, and the mean values ± SD from three independent experiments are shown. Asterisks indicate significant difference compared with silkworms on the normal diet (p < 0.05, Student’s t-test). The log-transformed values were used in the statistical analysis because they exhibited higher normality than the non-transformed values. E. Hemolymph samples were collected from silkworms (n = 4) fed a normal diet or diet containing heat-killed *P*. *aeruginosa* cells. The samples, containing 28 μg protein, were electrophoresed using 16.5% tricine sodium dodecyl sulfate-polyacrylamide gels. Proteins in the gels were then transferred to a membrane and analyzed by Western blotting using an anti-PP antibody. Asterisk indicates the active-form of PP. A representative result from two independent experiments is shown.

We then examined the molecular mechanism underlying the protective effect of oral administration of heat-killed *P*. *aeruginosa* cells against *P*. *aeruginosa* intra-hemolymph infection. We hypothesized that antimicrobial activity was induced against *P*. *aeruginosa* in the silkworm hemolymph. We collected hemolymph samples from silkworms orally administered heat-killed *P*. *aeruginosa* cells, and cultured living *P*. *aeruginosa* cells in the hemolymph samples. Compared to the control sample, the growth of *P*. *aeruginosa* in the hemolymph from silkworms administered the heat-killed *P*. *aeruginosa* was inhibited (Fig 5A). This finding indicates that antimicrobial activity against *P*. *aeruginosa* was induced in the silkworm hemolymph by oral administration of heat-killed *P*. *aeruginosa*.

### Expression of immune-associated genes by oral administration of heat-killed *P*. *aeruginosa*

Because silkworms orally administered heat-killed *P*. *aeruginosa* exhibited infection tolerance against *P*. *aeruginosa* and antimicrobial activity against *P*. *aeruginosa* was induced in the hemolymph of these silkworms, we hypothesized that heat-killed *P*. *aeruginosa* systemically activates silkworm immunity, resulting in the transcriptional activation of genes coding antimicrobial peptides. We measured the amounts of cecropin A and moricin mRNA in the midgut, fat body, and hemocytes of silkworms fed a diet with or without heat-killed *P*. *aeruginosa* cells. In the fat body and hemocytes, the amounts of cecropin A and moricin mRNA were greater in silkworms fed the diet containing the heat-killed *P*. *aeruginosa* cells than in silkworms fed the normal diet (Fig 5B and 5C). The amount of cecropin A mRNA was also increased in the midgut, whereas that of moricin was not (Fig 5B and 5C). These findings suggest that systemic activation of antimicrobial peptide expression occurs in silkworms orally administered heat-killed *P*. *aeruginosa* cells.

In insects, the expression of antimicrobial peptides is regulated by nuclear factor-kB, which is regulated by the I kappa B kinase (IKK) complex in the IMD pathway [30]. The expression of IKKγ, a component of the IKK complex, was upregulated in the fat body and hemocytes of silkworms orally administered heat-killed *P*. *aeruginosa* cells (Fig 5D). A recent report demonstrated that the insect cytokine paralytic peptide (PP) is activated by the injection of peptidoglycans, leading to the systemic activation of innate immune responses [31]. The amount of activated PP was increased in the hemolymph of silkworms orally administered heat-killed *P*. *aeruginosa* cells (Fig 5E). These findings suggest that oral administration of heat-killed *P*. *aeruginosa* cells activates the PP and IMD pathways, and results in the systemic expression of antimicrobial peptides.

## Discussion

**Fig 6:**
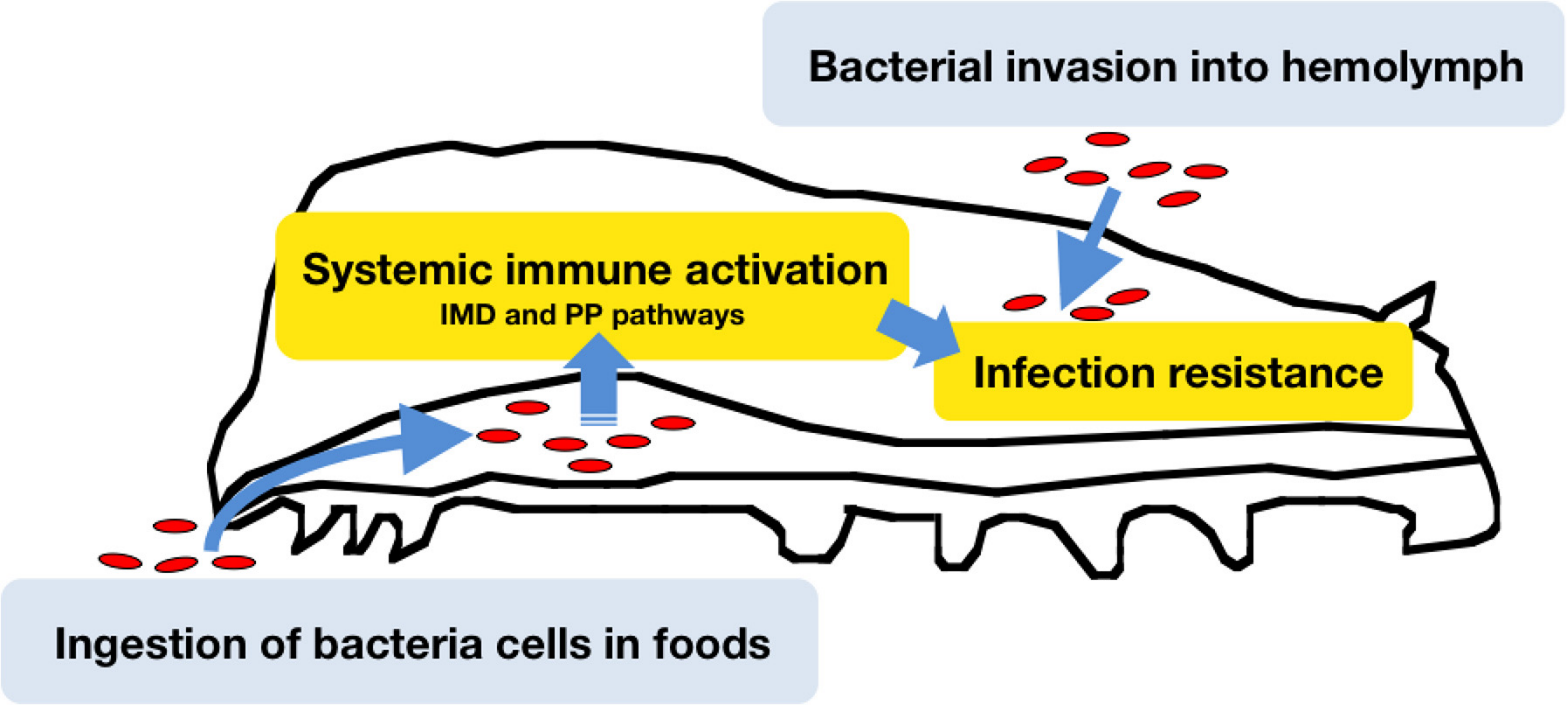
Model of primed immune responses triggered by ingestion of bacteria. Silkworms ingest bacteria with their food. The ingested bacteria activate systemic immune responses in the gut, which leads to tolerance against bacterial invasion into the hemolymph.

The findings of the present study demonstrated that oral administration of heat-killed microbes, conferred resistance of silkworms against *P*. *aeruginosa* infection in the hemolymph. Further, oral administration of heat-killed *P*. *aeruginosa* cells induced the expression of antimicrobial peptide genes in the midgut, fat body, and hemocytes of silkworms. In addition, IKKγ and PP, which activate the expression of antimicrobial peptides, were increased by oral administration of heat-killed *P*. *aeruginosa* cells, suggesting that the silkworms innate immune system responds to orally-ingested microorganisms, resulting in resistance against subsequent microbial infection in the hemolymph (Fig 6). This study is the first to demonstrate the physiologic significance of systemic immune activation by ingested bacteria in insects as a primed immune response.

The present study revealed that oral administrations of heat-killed *P*. *aeruginosa* cells, heat-killed *S*. *marcescens* cells, and heat-killed *C*. *albicans* cells had significant effects to protect silkworms against subsequent infection by *P*. *aeruginosa*. In contrast, heat-killed *S*. *aureus* cells did not protect silkworms against *P*. *aeruginosa* infection. This indicates that the primed immune response protecting silkworms against *P*. *aeruginosa* infection are triggered by ingestion of Gram-negative bacteria cells or fungal cells, but not by ingestion of Gram-positive bacteria. These findings are also consistent with our previous observation that injecting heat-killed *S*. *aureus* cells into silkworm hemolymph did not confer a protective effect against *E*. *coli* infection [23]. *S*. *marcescens* and *P*. *aeruginosa* infect various insects in natural environments [18, 32], whereas *S*. *aureus* is not a natural insect pathogen (the Ecological Database of the World’s Insect Pathogens) [33]. Insects may possess a sensitive immune-activation system for these naturally pathogenic Gram-negative bacteria.

This study demonstrated that oral administrations of peptidoglycans from *P*. *aeruginosa*, *S*. *aureus*, and *L*. *plantarum* showed the protecting effects against *P*. *aeruginosa* infection. We did not detect difference between the effect of peptidoglycans from *S*. *aureus* and *L*. *plantarum*, which contain Lys-type peptidoglycans and DAP-type peptidoglycans, respectively (p = 0.944). This indicates that silkworm immune system can recognize both the Lys-type and DAP- type peptidoglycans. Because the heat-killed *S*. *aureus* cells did not show the protecting effect, the *S*. *aureus* cellular peptidoglycan may have a different conformation from that of the purified peptidoglycan, which escapes the recognition by the silkworm immune system. Further investigations are necessary to answer why the silkworm immune system recognizes orally ingested heat-killed *P*. *aeruginosa* cells, but not the heat-killed *S*. *aureus* cells.

Both PP and IKKγ are suggested to be involved in the mechanism underlying immune activation by orally ingested heat-killed *P*. *aeruginosa* cells. Because PP does not increase the amount of IKKγ [31] and the activation of PP occurs within minutes by reactive oxygen species [34], the PP and IKKγ pathways may be independently activated by gut epithelial cells after the recognition of heat-killed *P*. *aeruginosa* cells to activate systemic production of antimicrobial peptides. Previous studies reported that hemocytes are involved in the primed immune responses in lower organisms [22]. In the woodlouse *Porcellio scaber*, an increasing number of phagocytosing hemocytes are involved in the primed immune response [35]. In mosquitoes *Anopheles gambiae*, the number of granulocytes increases to protect against parasite infection [20]. Further studies are needed to reveal the molecular mechanisms of microorganism recognition by gut epithelial cells and the involvement of hemocytes to lead systemic activation of immune reactions as a primed immunity.

Animals, from arthropods to humans, ingest many bacterial or fungal cells collaterally with their food sources. Humans, in addition, have developed fermentation techniques to make fermented food. Several insect species prefer ingesting microbes, such as the leaf-cutting ant, which cultivates fungus on plant leaves. The present study demonstrates a physiological significance of microbes present in animal food sources such that they activate immune systems to defend animals against potential microbial infection.

## Materials and Methods

### Bacteria or fungi

*P*. *aeruginosa* PAO1 strain was cultured in Luria-Bertani medium (1% Tryptone, 0.5% yeast extract, 1% NaCl) at 30°C. *S*. *aureus* NCTC8325-4 was cultured at 37°C in Tryptic Soy Broth medium (BD Bioscience). *S*. *marcescens* 2170 strain was cultured at 30°C in Brain-Heart Infusion medium (BD Bioscience). *C*. *albicans* ATCC10231 was cultured at 37°C in YPD medium. *L*. *plantarum* JCM1057 (Riken BRC, National BioResource Project of the MEXT, Japan) was cultured in MRS medium at 30°C.

### Insects

Eggs of *Bombyx mori* silkworms were purchased from Ehime-Sanshu (Ehime, Japan). The hatched silkworm larvae were fed an artificial diet containing antibiotics (Silkmate 2S, Nihon Nosan Corporation, Yokohama, Japan) up to the fifth instar larval stage. After the fifth instar stage, the silkworms were fed an antibiotic-free diet (Katakura Industries, Tokyo, Japan) [36].

### Heat treatment of bacteria or fungi

The overnight culture of each bacterium or fungus was centrifuged (12,000 *g*, 4°C, 10 min), and the pellets were re-suspended in saline. The suspensions were autoclaved at 121°C for 15 min. Autoclaved samples were centrifuged (12,000 *g*, 4°C, 10 min) and the supernatants were removed. The samples were stored at -20°C.

### Oral administration of heat-killed bacteria to silkworms

A fifth instar silkworm larva consumes ~2.3 g of artificial diet over 2 d. An artificial diet containing heat-killed bacteria or fungi was prepared by mixing 23 g of antibiotic-free artificial diet and heat-killed bacterial or fungal suspension from overnight cultures. The actual volume of the culture for each experiment is listed in S1 Table. The artificial diet with or without heat-killed bacteria cells was fed to fifth instar silkworms from day 1 to day 3 of the fifth instar larval stage.

### Intra-hemolymph infection of silkworms

After feeding on the artificial diets for 2 d, silkworms were injected into the hemolymph with living bacteria cells (50 μL) using a 1-mL syringe equipped with a 27-gauge needle (Terumo) [37]. Overnight cultures of *P*. *aeruginosa*, *S*. *aureus*, and *C*. *albicans* were diluted 10^3^-fold, 30-fold, and 10-fold, respectively, and used for infection. The silkworms were then incubated in a plastic cage at 27°C and survival was assessed.

### Extraction of lipopolysaccharides and peptidoglycans from *P*. *aeruginosa*

Lipopolysaccharides were extracted by the hot-phenol method [38]. Fourteen liters of *P*. *aeruginosa* culture was centrifuged (12,000 *g*, 10 min, 4°C) and washed with saline. The pellets were re-suspended in acetone, centrifuged (16,200 *g*, 10 min, 4°C), and dried. The dried sample was suspended in 65°C water and sonicated. An equal volume of 90% phenol (preheated to 65°C) was added and incubated at 65°C for 30 min. The centrifuged supernatant was mixed again with equal volume of 90% phenol (preheated to 65°C) and incubated for 30 min. The centrifuged sample was dialyzed against water, frozen at -20°C, and dried using a freeze dryer. The sample was suspended in 0.5 M NaCl and mixed with 1.5 volume of 2% Cetavlon (cetyltrimethylammonium bromide)/0.5 M NaCl. The centrifuged supernatant was frozen at -20°C and dried using a freeze dryer. The sample was suspended in 0.5 M NaCl and mixed with 10 volumes of ethanol and stirred at 4°C for 2 h. The sample was centrifuged (16,200 *g*, 10 min, 4°C) and the precipitate was suspended in water. The sample was dialyzed against water, frozen at -20°C, dried using a freeze dryer, resulting in lipopolysaccharide fraction. We measured its dry weight to quantify the lipopolysaccharide sample. The sample was suspended in saline before use.

Peptidoglycans were extracted as described previously [39]. Two liters of *P*. *aeruginosa* culture was centrifuged (12,000 *g*, 10 min, 4°C) and washed with saline. The pellets were re-suspended in water and autoclaved for 15 min at 121°C. Samples were washed with water and acetone three times each, and then dried at room temperature. The dried powder was suspended in water and fractured using a French press (Thermo Fisher Scientific). The fractured sample was washed three times with water, then dried at room temperature, and suspended in phosphate-buffered saline. The suspension was treated overnight with DNaseI (Takara) and RNaseA (Sigma) at 37°C, followed by treatment with trypsin (Nacalai) at 37°C overnight. The sample was then washed three times with water, suspended in 5% trichloroacetate, and incubated overnight at 27°C. After 1 h at 4°C, the sample was centrifuged (16,200 *g*, 4°C, 10 min) and the pellet was washed three times with water and acetone, dried at room temperature, and stored at -20°C. We measured its dry weight to quantify the peptidoglycan sample. The prepared peptidoglycans sample was suspended in phosphate-buffered saline and treated by sonication before use.

### Quantitative reverse transcription-polymerase chain reaction

Silkworms on day 1 of the fifth instar stage were fed an artificial diet containing heat-killed *P*. *aeruginosa* cells and incubated for 2 d at 27°C. Total RNA from hemocytes, midgut, and fat body of the silkworms was extracted using an RNeasy Mini Kit (Qiagen), according to the manufacturer’s protocol. After digestion of the contaminated genomic DNA by RQ1 RNase-free DNase (Promega), the RNA was reverse-transcribed to cDNA using TaqMan reverse transcriptase (Applied Biosystems). Quantitative real-time polymerase chain reaction was performed using oligonucleotide primers (Table 1), cDNAs as a template, and FastStart SYBR Green Master (Roche).

**Table 1:** Primers used in this study.

Target	Primer	Sequence (5'-3')
*elongation factor 2*	F-ef2	GTGCGAGAGCCGGAGAGAC
	R-ef2	CGAAGAACATAGAGATGGCCG
*cecropin A*	F-cecA	TTGAGCTTCGTCTTCGCGTT
	R-cecA	TTGCGTCCCACTTTCTCAATT
*moricin*	F-mor	CCGCTCCAGCAAAAATACCT
	R-mor	TTGAAAACATCGTTGGCTGT
*IKKγ*	F-IKKg	GACGACGACACCATGAA
	R-IKKg	AACTATATGCTCCAGGG

### Detection of activated PP in the silkworm hemolymph

Silkworms were fed an artificial diet with or without heat-killed bacteria. Prolegs were dissected to collect the hemolymph. Immediately after hemolymph collection, the hemolymph samples were boiled for 5 min. The supernatant of the boiled samples was centrifuged (10,000 *g*, 5 min) and analyzed by Western blotting. Synthesized active-form PP was used as a control [34]. Protein samples were electrophoresed using 16.5% tricine-sodium dodecyl sulfate-polyacrylamide gels, and transferred to an Immobilon-P polyvinylidene difluoride membrane (Millipore). The membrane was hybridized with 1:6000 diluted anti-PP antibody suspended in a blocking solution, and then reacted with 1:5000 diluted anti-rabbit immunoglobulin G from donkey linked with horseradish peroxidase (Amersham Biosciences). The membrane was treated with Western Lightning Chemiluminescence Reagent Plus (PerkinElmer Life Sciences), and luminescence signals were detected on autoradiography film (Hyperfilm-ECL, Amersham Biosciences, UK).

### Statistical analysis

A statistical summary of all infection experiments in this study is shown in S1 and S2 Tables. The replicates for each experiment were then combined into a single analysis. To test the difference in survival curves, we performed log-rank tests using R ver.2.15.3 on Mac OS X. The package “survival” was used to perform a log-rank test. We applied Bonferroni’s correction to test the significance level of the differences of multiple samples. To test the difference in mean values, we performed Student’s t-test using Microsoft Excel 2011 for Mac OS.

## Supporting Information

S1 Fig Resistance of silkworms orally administered with heat-killed cells of various microorganisms against microbial infection. Silkworms were fed a normal diet or a diet containing heat-killed microbial cells for 2 d, and then injected with living microbial cells. Experimental conditions and statistical analysis for each experiment are presented in S1 Table.
(TIF)

S2 Fig Resistance of silkworms orally administered with lipopolysaccharide or peptidoglycan against *P*. *aeruginosa* infection. Silkworms were fed a normal diet or a diet containing lipopolysaccharide or peptidoglycan for 2 d, and then injected with living *P*. *aeruginosa* cells. Experimental conditions and statistical analysis for each experiment are presented in S2 Table.
(TIF)

S1 Table Summary of experiments using heat-killed bacterial or fungal cells. (DOCX)

S2 Table Summary of experiments using lipopolysaccharide or peptidoglycan samples. (DOCX)
