# Supplementary figures and images for: Primed Immune Responses Triggered by Ingested Bacteria Lead to Systemic Infection Tolerance in Silkworms
Source: PLoS One. 2015 Jun 24;10(6):e0130486. doi: 10.1371/journal.pone.0130486 (PMC4479504; doi:10.1371/journal.pone.0130486)

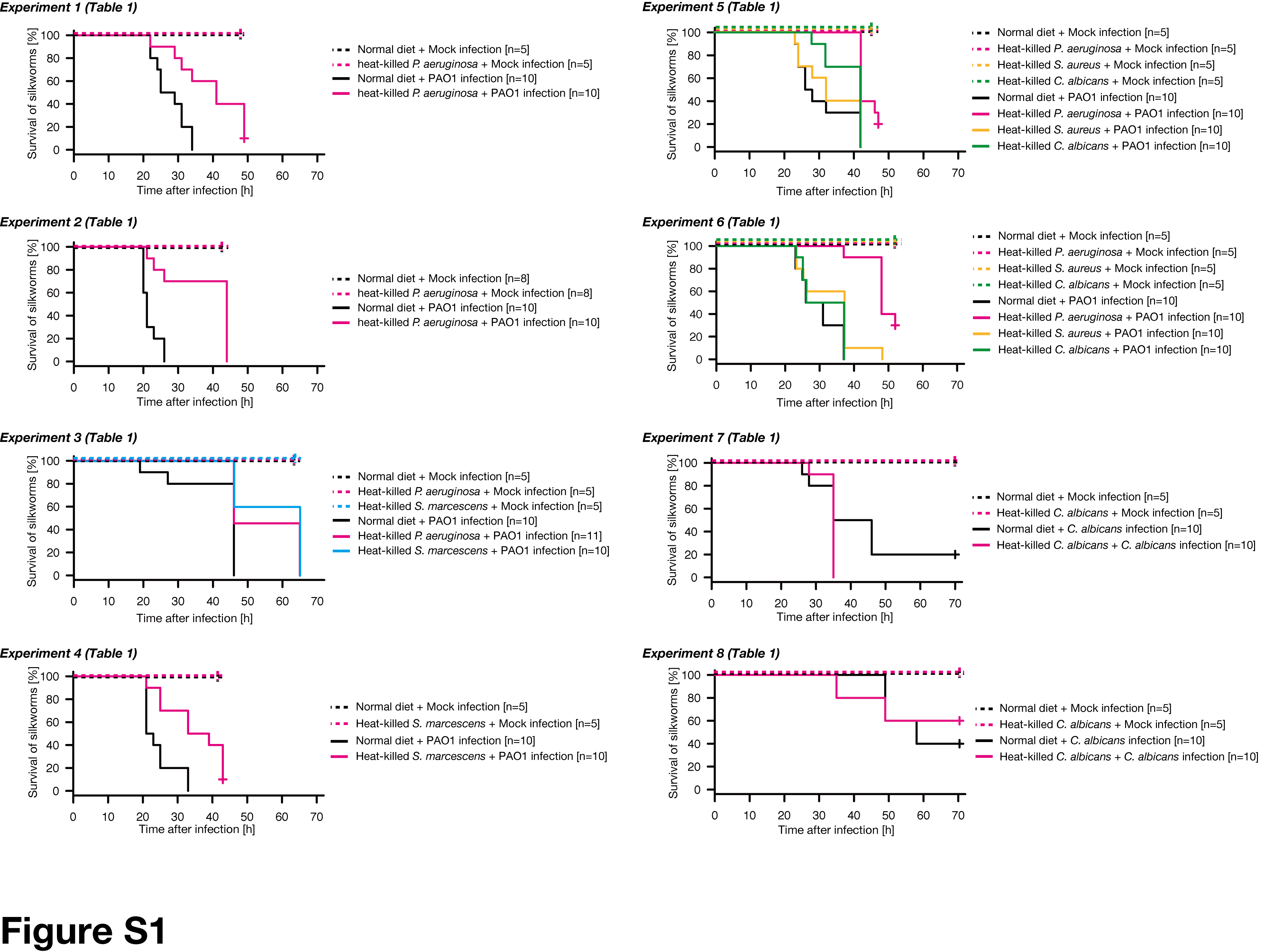

Supplement: S1 Fig — Silkworms were fed a normal diet or a diet containing heat-killed microbial cells for 2 d, and then injected with living microbial cells. Experimental conditions and statistical analysis for each experiment are presented in S1 Table. (TIF) [file pone.0130486.s001.tif]

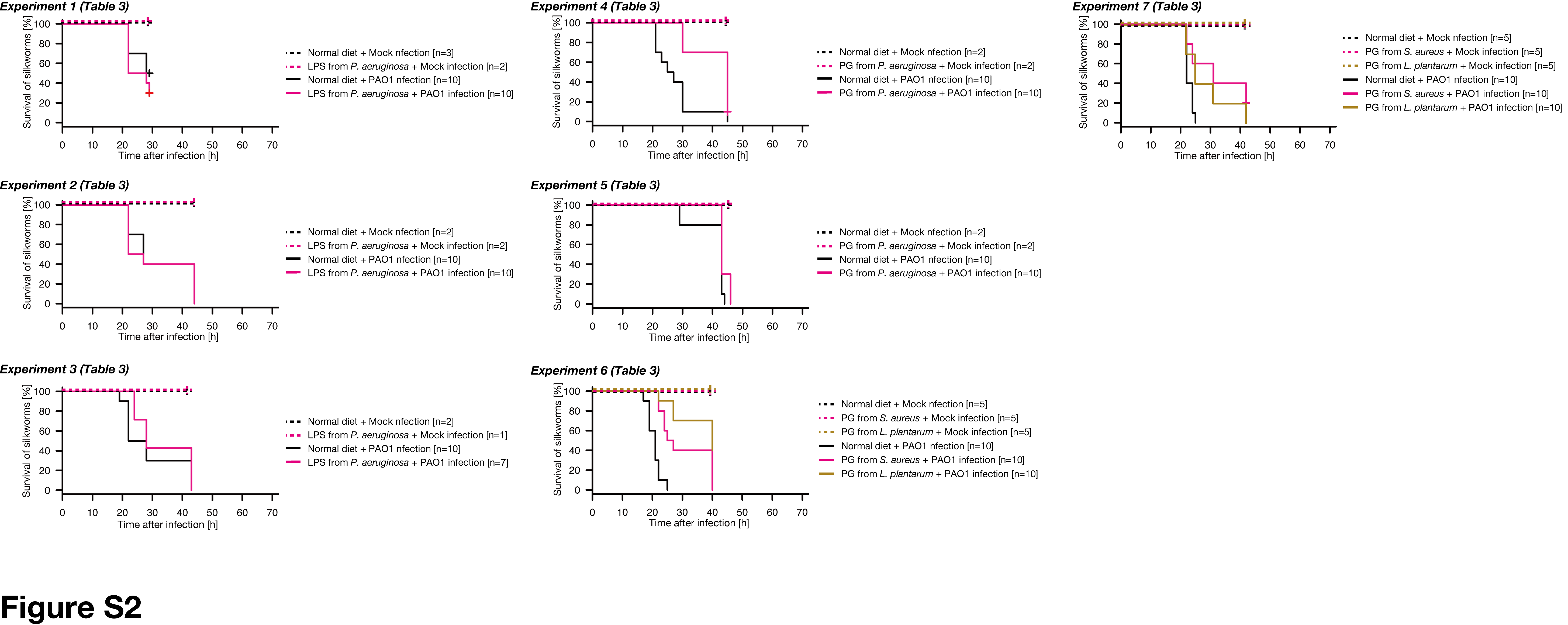

Supplement: S2 Fig — Silkworms were fed a normal diet or a diet containing lipopolysaccharide or peptidoglycan for 2 d, and then injected with living P. aeruginosa cells. Experimental conditions and statistical analysis for each experiment are presented in S2 Table. (TIF) [file pone.0130486.s002.tif]
