# Supplementary material for: Primed Immune Responses Triggered by Ingested Bacteria Lead to Systemic Infection Tolerance in Silkworms
Source: PLoS One. 2015 Jun 24;10(6):e0130486. doi: 10.1371/journal.pone.0130486 (PMC4479504; doi:10.1371/journal.pone.0130486)
Supplement: S1 Table — (DOCX) [file pone.0130486.s003.docx]

**Table S1. Summary of experiments using heat-killed bacterial or fungal cells.**

| Exp. | Fed sample | Dose | Infection | n | | Effect | P-value | SL |
| --- | --- | --- | --- | --- | --- | --- | --- | --- |
|  | (mixed in the diet) | (ml/larva) |  | mock | infected |  |  |  |
| 1 | Heat-killed *P. aeruginosa* | 66 | *P. aeruginosa* | 5 | 10 | + | 3.92E-03 | 0.05 |
|  | Normal Diet | 0 | *P. aeruginosa* | 5 | 10 |  |  |  |
| 2 | Heat-killed *P. aeruginosa* | 33 | *P. aeruginosa* | 8 | 10 | + | 3.77E-04 | 0.05 |
|  | Normal Diet | 0 | *P. aeruginosa* | 8 | 10 |  |  |  |
| 3 | Heat-killed *S. marcescens* | 67 | *P. aeruginosa* | 5 | 10 | + | 2.97E-03 | 0.05/2 |
|  | Heat-killed *P. aeruginosa* | 37 | *P. aeruginosa* | 5 | 11 | + | 8.18E-03 | 0.05/2 |
|  | Normal Diet | 0 | *P. aeruginosa* | 5 | 10 |  |  |  |
| 4 | Heat-killed *S. marcescens* | 67 | *P. aeruginosa* | 5 | 10 | + | 3.54E-03 | 0.05 |
|  | Normal Diet | 0 | *P. aeruginosa* | 5 | 10 |  |  |  |
| 5 | Heat-killed *P. aeruginosa* | 67 | *P. aeruginosa* | 5 | 10 | + | 5.70E-04 | 0.05/3 |
|  | Heat-killed *S. aureus* | 67 | *P. aeruginosa* | 5 | 10 | - | 0.561 | 0.05/3 |
|  | Heat-killed *C. albicans* | 67 | *P. aeruginosa* | 5 | 10 | - | 0.0311 | 0.05/3 |
|  | Normal Diet | 0 | *P. aeruginosa* | 5 | 10 |  |  |  |
| 6 | Heat-killed *P. aeruginosa* | 67 | *P. aeruginosa* | 5 | 10 | + | 2.46E-05 | 0.05/3 |
|  | Heat-killed *S. aureus* | 67 | *P. aeruginosa* | 5 | 10 | - | 0.234 | 0.05/3 |
|  | Heat-killed *C. albicans* | 67 | *P. aeruginosa* | 5 | 10 | - | 0.495 | 0.05/3 |
|  | Normal Diet | 0 | *P. aeruginosa* | 5 | 10 |  |  |  |
| 7 | Heat-killed *C. albicans* | 61 | *C. albicans* | 5 | 10 | - | 0.104 | 0.05 |
|  | Normal Diet | 0 | *C. albicans* | 5 | 10 |  |  |  |
| 8 | Heat-killed *C. albicans* | 60 | *C. albicans* | 5 | 10 | - | 0.635 | 0.05 |
|  | Normal Diet | 0 | *C. albicans* | 5 | 10 |  |  |  |

Silkworms were fed heat-killed bacteria or fungi, and infected with bacteria or fungi in the hemolymph. Survival of the silkworms was monitored. Experimental condition and statistical information for each infection experiment are shown. Experiments with identically fed samples and infected pathogens are listed in the same color. Fed samples, sample fed to silkworms; Dose, volume of full growth equivalent to the sample fed to a silkworm larva; Infection, bacteria or fungi used to infect silkworms after 2-d ingestion of the sample; n, number of silkworms used in each experiment; mock, number of silkworms injected with saline after the 2-d ingestion; infected, number of silkworms injected with living microbial cells after the 2-d ingestion; Effect, “+” indicates the sample prolonged the survival of silkworms, and “-“ indicates no such effect was observed in each trial; P-value, difference in the survival curve from control (silkworms fed normal diet) was tested by log-rank test and the p-values are listed; SL, significance levels for each experiment corrected by Bonferroni’s correction to test multiple samples. No death was observed in mock-infected silkworms. The survival curve for each experiment is shown in Figure S1.
