# Supplementary material for: Primed Immune Responses Triggered by Ingested Bacteria Lead to Systemic Infection Tolerance in Silkworms
Source: PLoS One. 2015 Jun 24;10(6):e0130486. doi: 10.1371/journal.pone.0130486 (PMC4479504; doi:10.1371/journal.pone.0130486)
Supplement: S2 Table — (DOCX) [file pone.0130486.s004.docx]

**Table S2. Summary of experiments using lipopolysaccharide or peptidoglycan samples.**

| Exp. | Fed sample | Dose | Infection | n | | Effect | P-value |  |
| --- | --- | --- | --- | --- | --- | --- | --- | --- |
|  |  | (mg/larva) |  | mock | infected |  |  |  |
| 1 | LPS from *P. aeruginosa* | 6.7 | *P. aeruginosa* | 2 | 10 | - | 0.582 |  |
|  | Normal Diet | 0 | *P. aeruginosa* | 3 | 10 |  |  |  |
| 2 | LPS from *P. aeruginosa* | 3.8 | *P. aeruginosa* | 2 | 10 | - | 0.812 |  |
|  | Normal Diet | 0 | *P. aeruginosa* | 2 | 10 |  |  |  |
| 3 | LPS from *P. aeruginosa* | 2.5 | *P. aeruginosa* | 1 | 7 | - | 0.364 |  |
|  | Normal Diet | 0 | *P. aeruginosa* | 2 | 10 |  |  |  |
| 4 | PG from *P. aeruginosa* | 15 | *P. aeruginosa* | 2 | 10 | + | 1.37E-03 |  |
|  | Normal Diet | 0 | *P. aeruginosa* | 2 | 10 |  |  |  |
| 5 | PG from *P. aeruginosa* | 16 | *P. aeruginosa* | 2 | 10 | + | 0.0374 |  |
|  | Normal Diet | 0 | *P. aeruginosa* | 2 | 10 |  |  |  |
| 6 | PG from *S. aureus* | 41 | *P. aeruginosa* | 5 | 10 | + | 4.00E-04 |  |
|  | PG from *L. plantarum* | 41 | *P. aeruginosa* | 5 | 10 | + | 1.75E-05 |  |
|  | Normal Diet | 0 | *P. aeruginosa* | 5 | 10 |  |  |  |
| 7 | PG from *S. aureus* | 41 | *P. aeruginosa* | 5 | 10 | + | 3.77E-03 |  |
|  | PG from *L. plantarum* | 41 | *P. aeruginosa* | 5 | 10 | + | 7.09E-03 |  |
|  | Normal Diet | 0 | *P. aeruginosa* | 5 | 10 |  |  |  |

Silkworms were fed lipopolysaccharide (LPS) or peptidoglycan (PG) mixed with a normal diet, and infected with *P. aeruginosa* in hemolymph. Survival of the silkworms was monitored. Experiments with identically fed samples and infected pathogens are listed in the same color. Fed sample, sample that was fed to silkworms; Dose, dose of the sample (dry weight); Infection, bacteria used to infect silkworms after 2-d ingestion of the sample; n, number of silkworms used in each experiment; mock, number of silkworms injected with saline after the 2-d ingestion; infected, number of silkworms injected with living microbial cells after the 2-d ingestion; Effect, “+” indicates the sample prolonged the survival of silkworms, and “-“ indicates no such effect was observed in each trial; P-value, the difference in the survival curve from control (silkworms fed with normal diet) was tested by log-rank test. No death was observed in mock-infected silkworms. The survival curve for each experiment is shown in Figure S2.
